# Avoiding digital marketing analytics myopia: revisiting the customer decision journey as a strategic marketing framework

**DOI:** 10.1057/s41270-020-00098-0

**Published:** 2021-01-08

**Authors:** Matthew D. Vollrath, Salvador G. Villegas

**Affiliations:** 1 Department of Economics and Business, Ohio Wesleyan University, 61 S. Sandusky St, Delaware, OH 43015 USA; 2 School of Business, Northern State University, Aberdeen, USA

**Keywords:** Marketing myopia, Digital marketing analytics, Consumer decision journey, Integrated marketing communications

## Abstract

The use of analytics data in digital marketing has had a profound impact on the way marketers create consumer relationships and how firms make decisions. However, the marketing analytics literature offers little guidance on how digital marketing analytics tools should be selected and leveraged in service to the firm’s overall strategy. Foundational marketing theory and research concerning the origin of consumer value and the primary importance of the consumer decision journey to strategy formation offer a pathway to evaluating digital marketing tools and analysis in a strategic and theoretically sound manner. This paper builds on seminal marketing thought to propose a conceptual framework that places use of digital marketing analytics tools and channels in the context of a firm’s marketing plan. The framework has diverse applications across many industries and platforms and can help markers avoid falling victim to digital marketing analytics myopia, even as evolving technologies and broader societal forces like the response to Covid-19 accelerate the digitalization of marketing.

## Introduction

How customers experience a brand is increasingly taking place online. It is estimated that e-commerce accounted for over 14% of global retail sales in 2019 and that it will account for 22% of global retail sales by 2023 (eMarketer 2019). COVID-19 may accelerate this growth, with 48% of consumers reporting in May 2020 that the virus had caused them to purchase products online that they would have usually purchased in stores (Numerator Intelligence 2020). Digital retail sales are quickly becoming a necessary sales channel for consumers and may no longer be seen as merely an alternative to traditional brick and mortar shopping. At the same time, marketers reported allocating 50.1% of their budgets to digital marketing channels in 2019 and are forecasting to spend 60.5% of their marketing budgets on digital initiatives by 2023 (eMarketer 2020). In this digitally driven environment marketing analytics are essential and manifold (Saura et al. 2017). There is an abundance of marketing literature exploring practical aspects of marketing analytics ranging from basic definitions (Iacobucci et al. 2019), to specific applications (Mikalef et al. 2018), and adoption within a firm (Branda et al. 2018) but relatively little has been written about analytics integration with marketing theory (Iacobucci et al. 2019). It has been observed in this journal that “metrics and data are empty shells without proper theories and interpretations behind them (Krishen and Petrescu 2017, p. 117).” What is missing from the marketing analytics literature is a conceptual yet functional framework that is grounded in seminal marketing thought, connecting the selection and use of marketing analytics to an organization's comprehensive marketing strategy. This paper will briefly review how marketing literature has generally approached the topic of marketing analytics and consider how marketing research combined with theory suggests directions for a conceptual and strategic digital marketing analytics framework. This paper then proposes such a framework and argues that even as emerging technologies, new marketing channels, and the realities of COVID-19 accelerate the digitalization of marketing activities, a customer journey focused approach to marketing can offer clarity about what data is strategically valuable across diverse industries and circumstances.

## Literature review

### A need for strategic integration

The concept of marketing analytics, defined by Iacobucci et al. (2019) as “the study of data and modeling tools used to address marketing resource and customer-related business decisions” (p. 155), has been present in the literature and industry since around the beginning of the twentieth century. It was not until the advent of the internet, and with it, technologies such as CRMs and search engines, that marketing analytics emerged as the field we know today (Wedel and Kannan 2016). Since that time, interest in marketing analytics has grown rapidly (Petrescu and Krishen 2017) as researchers explored the application of diverse marketing analytics techniques across diverse industries. This growth was largely driven by the exponential growth of available data, and marketers’ efforts to answer the basic question of *what to do with it all?* (Krishen and Petrescu 2017). Grappling with that question has produced research that focused on the *how* and *what* of marketing analytics, offering definitions, techniques, applications, and assessments of marketing analytics’ impact (Wedel and Kannan 2016). What was and remains neglected in the literature, is a guiding consensus about which of the myriad techniques are most valuable (Saura et al. 2017), and how digital marketing analytics can be effectively integrated into an organization’s overall marketing strategy (Kingsnorth 2019; Iacobucci et al. 2019). Indeed, while the use of data tools has been welcomed in the business world as a possible solution to most problems, their implementation is often deemed a failure, and their results are seen as disappointing (Tabesh et al. 2019). From a marketing perspective, key reasons for these failures are reliance on confusing and disparate planning frameworks (McTigue 2019), and focusing on tools, specific marketing metrics or financial returns instead of the consumer’s needs (Dimitriadis et al. 2018; Kaushik 2015; Grigsby 2015). In other words, these failures are strategic failures.

### Marketing strategy and consumer needs

The broader marketing literature has long grappled with reconciling disconnections between marketing strategy and tactics, and more importantly, disconnections between marketing strategy and the consumer. Theodore Levitt’s (2016) seminal 1960 article, *Marketing Myopia*, diagnosed marketers’ primary strategic problem as operating from a perspective that prioritized the firm's products and goals over consumers’ needs:

The usual result of this narrow preoccupation with so-called concrete matters is that instead of growing…the product fails to adapt to the constantly changing patterns of consumer needs and tastes…The industry has its eyes so firmly on its own specific product that it does not see how it is being made obsolete. (p. 45).

By themselves and without customer focus, marketing analytics can lead to the very same trap that Levitt (2016) described. Wells Fargo’s use of analytics offers a prime example of this problem. In the years leading up to an ethics crisis that would cost the bank billions in fines and inflict enduring reputational damage (Eisen 2020), Wells Fargo was known for its highly advanced use of analytics throughout its operations. However, these analytics tools were not employed to understand and create value from the consumer’s perspective, but rather to create value from the *firm's perspective*. Wells Fargo leadership set a customer relationship goal of eight accounts per customer, leveraged analytics tools toward that goal, and two million fake/unauthorized customer accounts were the end result (Ali et al. 2018). When the focus of analytics is boosting profit from existing products and services, firms are effectively deciding that what consumers need does not matter. Consumers must buy more of what is being sold, regardless of their unique needs and circumstances.

The marketing analytics myopia displayed by Wells Fargo is not unique and manifests itself wherever a marketer or firm leader is stubbornly committed to any singular metric or dashboard. Kaushik (2015) described how digital marketing campaigns are destined to fail when marketers fixate on a key performance indicator (KPI) such as click thru rate (CTR), measuring campaigns against it regardless of customer segment or location in their buying journey. An antidote to these problems is a focus on creating customer value conceptualized through a consumer decision journey (Edelman 2010; Rust et al. 2010).

### Consumer decision journeys

#### AIDA

The idea that consumers experience the buying process as a journey can be traced as far back as 1898, when salesman and advertising pioneer Elmo St. Lewis proposed the now famous Awareness, Interest, Desire, Action (AIDA) framework (Strong 1925). St. Lewis’ central insight was that consumers need to receive different messages about a product at different times, moving along a linear path with specific steps of awareness, interest, and desire which culminate into action. The AIDA model is foundational to the field of marketing and advertising, in part because its conceptualization of marketing/advertising specific activities facilitates measurement as consumers move from one stage of their cognitive and affective journey to the next (Wijaya 2012). This concept translates neatly into a funnel metaphor in which many possible consumers are narrowed to fewer with interest, still fewer with desire, and ultimately fewer still who become customers. Digital marketers are typically adept at building effective and measurable strategies around this linear conception of the consumer decision journey (Kingsnorth 2019).

#### The modern consumer decision journey

Over the last decade, many marketing researchers and practitioners have shifted away from the traditional AIDA model of thinking, toward a model that emphasizes the importance of consumer relationships (McTigue 2019). This shift seeks to account for the fact that people do not just buy brands as isolated transactions, but buy them based on a personal perception of value formed by the totality of their experiences with the brand. This concept postulates that when a consumer becomes a customer, the relationship they form with the brand becomes part of the total value that the brand offers (Edelman 2010). This aspect of consumer behavior is neglected in both linear AIDA and funnel models, but included in a circular model of the consumer decision journey first proposed by McKinsey consultants (Court et al. 2009).

The McKinsey model sees the consumer decision journey as a four-part directional process in which the consumer: (1) begins with a list of brands they intend to consider, (2) adds or subtracts brands to the list as they evaluate what they want, (3) makes a purchase, and (4) builds expectations based on their experience with the product or service to inform future behavior (Court et al. 2009). When consumers are satisfied with the total value that a firm has provided throughout these four stages, they are likely to skip steps one and two of their buying journey the next time a buying need arises and go directly to step three—making a purchase.

Subsequent research has suggested a multitude of slight variations to the stages and terminology of this model (e.g. Wolny and Charoensuksai 2014; Young 2014; Kaushik 2015; Kotler et al. 2016; Katz 2017; Kingsnorth 2019), but at a high-level, these stages are accepted as the modern conception of the consumer decision journey (McTigue 2019). One variation on the model that stands out for its simplicity was proposed by Avinash Kaushik, Marketing Evangelist for Google. Kaushik (2013, 2015) conceptualized the four stages of the journey as See (awareness), Think (evaluation), Do (purchase), and Care (managing the post-purchase experience), and created the framework that is used internally at Google (Eriksson 2015). The simplicity of this model lends itself to quick understanding and easy translation to diverse business situations, an essential attribute for any model's use and adoption (McTigue 2019). The one-word verb descriptions of each stage orient the marketer to a consumer-centric perspective, and correspond to stages 1 through 4 of the McKinsey model. It is also a useful model to adopt in the context of a discussion of digital marketing analytics because as it was specifically developed for digital marketing (Kaushik 2013).

Whatever the specific model, the modern consumer decision journey implies that marketing strategies, tactics, and measurements should be aligned with the needs and behaviors of consumers at each of the decision journey stages (Kingsnorth 2019; Malthouse et al. 2019). This pushes the consumer decision journey beyond abstraction and into the world of practical application—the world of digital marketing analytics.

#### Psychological stages vs. brand encounters

It is important to note the difference between the consumer decision journey as a framework for understanding the psychological stages that consumers go through when making a purchase (Court et al. 2009), in contrast to a consumer journey as a map of brand encounters that consumers experience as they consider and ultimately complete a purchase (Vakulenko et al. 2019). The latter represents actual experiences or destinations that a consumer navigates in the process of making a purchase, while the former represents a state of mind that a consumer may hold across multiple stages of their decision journey. For example, a customer in the post-purchase stage of the consumer decision journey may travel a journey *map* that includes a visit to the product support website followed by viewing product tutorials on the company’s YouTube page. Both of these journey map destinations are encompassed by the fourth stage of the customer decision journey in which customers build expectations that will inform their future behavior. This distinction is significant because the journey map invariably represents occasions when a consumer is seeking to fulfill a functional need (e.g., how can I fix the problem I’m experiencing with this product?), while the decision journey represents the changing psychological needs that a consumer is seeking to fulfill through their interactions with the company (e.g., do I believe the company I purchased from cares about me and its brand is aligned with my conception of value?). Meeting functional needs may satisfy a customer, but it will not be enough to create customer loyalty. Consumer behavior research is clear that brand loyalty requires addressing emotional needs as well as functional expectations (Johansson and Carlson 2015).

### Segmentation

An implication of the consumer decision journey (See-Think-Do-Care), is that segmentation based on consumer behavior should be the starting point for effective marketing. Describing and reacting to consumer behavior is at the heart of marketing analytics, but attention to consumer behavior will lead marketers in diverse directions depending on the behavior in focus. Absent a framework for strategic segmentation, analytics may lead marketers astray, or at least produce suboptimal results. The consumer decision journey pushes marketers to embrace several basic strategic concepts.

#### Layers of segmentation

Consumers are diverse and behave differently based on their different needs (Peter and Olson 2010) and where they are in the consumer decision journey (Edelman and Singer 2015). Salient segments for an athletic apparel company, for example, might include consumers who use the product when they run, and those who use the product when they play basketball. Some consumers in the runners segment have never bought from the firm before, and others are repeat customers. One layer of segmentation then is the consumer’s location within the consumer decision journey, and another layer is how the consumer will ultimately use the product, that is, the problem the product solves (Christensen et al. 2005). Both layers of segmentation are defined by consumer behavior, and both layers have implications for which marketing message and platform should be employed for the target consumer (Young 2014; Hughes et al. 2019; Kingsnorth 2019). Ignoring this segment or the stage will result in marketing that does not fit the consumer.

One potential digital marketing analytics segmentation pitfall is an uneven use of analytics at different stages of the consumer decision journey. It is easy to measure and react to metrics such as CTR, as a natural application of digital marketing analytics is to use that data to maximize the efficiency of a specific campaign. However, maximizing a campaign on a single metric may not be consistent with the broader goals of the firm. The classic example is the parable of the AI program tasked with ensuring an office never ran out of paperclips. It did so by gaining control of the systems and organizations throughout the world, while leveraging the planet's resources to produce and protect paperclips (Sterne 2017). In the context of marketing, a strategy can become subject to ineffective tactics when it is matched with an inappropriate stage of the consumer decision journey. CTR will be nearly nonexistent at the See stage, and making marketing choices according to this metric will ignore the needs of that segment (Kaushik 2013). Maximizing based solely on CTR could produce impressive results at the Do stage, and also post-purchase cognitive dissonance that undermines the goals of the Care stage (Johansson and Carlson 2015).

The central assumption of the consumer decision journey framework is the goal of creating and maintaining customers who are loyal brand advocates (Edelman 2010; Rust et al. 2010). This is different from achieving a financial benchmark or hitting specific KPIs. Marketers may choose financial benchmarks and specific KPIs to help guide efforts at each stage of the consumer decision journey, but these should be subject to the overarching goals of loyalty and advocacy. This is a consumer-centric perspective: consumers will begin and continue their relationship with the firm because it consistently creates value for them (Levitt 1960; Sheth et al. 2000). Digital marketing analytics should help the firm create value for consumers by (1) producing insights about what fundamental problem consumers are trying to solve with the product, which is the source of its value according to Levitt (2016), and (2) connecting consumers to what they want (Hollebeek and Macky 2019).

### Mapping consumer journeys

A starting point for both of these value-based objectives is mapping the consumer journey. All consumers move through the consumer decision journey (See, Think, Do, Care), but may experience a variety of sequences of brand encounters. Mapping seeks to describe how consumers generally, and as salient segments (sometimes referred to as personas in the context of consumer journey mapping), typically navigate this process and why (Lemon and Verhoef 2016). Marketers can gain insight into these questions through methods that could include personal shopping diaries (Wolny and Charoensuksai 2014), personal interviews (Micheaux and Bosio 2019), customer surveys (De Keyser et al. 2015), combining third party socioeconomic and demographic data with customer purchase histories (Faulds et al. 2018), analyzing Google search data (Rennie et al. 2020), leveraging website analytics (Google Marketing Platform 2018), and creating blueprints of internal workflows (Birtel et al. 2016). Whatever the method, the goal is the same: to create a map connecting relevant marketing channels and consumer experiences to each stage of the consumer decision journey. It is critical for marketers to recognize that no single method of researching consumer decision journeys is likely to yield a complete picture of consumers' journey maps. Website analytics can show how consumers arrived at and moved through a firm's website, but offers little insight into the See stage of their journey. Blueprints of internal workflows can help a firm understand how consumers interact with its various departments, but this will be focused on the Do and Care stages of the journey. Qualitative research may address each stage of the journey, but findings and conclusions are inherently limited by the size of the study. Complete consumer decision journey mapping will likely require the use of multiple research methods.

## Application

Consumer journey mapping is valuable to the extent that it is viewed as a tool for understanding a consumer's need at each stage of their decision journey. When marketers lose sight of the journey map as a means for understanding consumer needs, the points of the map become just another misleading analytics metric. The firm's goal is not, for example, to ensure that e-commerce delivery times meet a certain threshold or trend in a certain direction. The goal is to ensure that the customer’s needs in the Care stage of their journey are met. This is bigger than any single metric, and maintaining this level of strategic focus throughout an organization requires a strategic framework for the selection and analysis of marketing analytics.

To this end, we propose the conceptual framework shown in Table 1: Using the Consumer Decision Journey for Strategic Selection of Digital Marketing Analytics Tools. The framework is built around Kaushik's (2015) simple See, Think, Do, Care conception of the consumer decision journey, which itself is rooted in the McKinsey model (Court et al. 2009). To use the framework, a marketer will consider the consumer decision journey of a specific segment, moving left to right across the table, selecting an appropriate tool for each column and stage (row). The first column of the framework lists the See, Think, Do, Care psychological stages of the consumer's decision journey, and the last (fourth) column of the table lists their corresponding measurable consumer behavior outcomes (as described in the McKinsey model), which will be practically defined differently by each firm. In between, column two focuses the marketer on Levitt's (2016) and Christensen et al.'s (2005) admonition to design offerings around the consumer's conception of value (solving their problem). This column acknowledges that understanding what constitutes value for a consumer must begin with market research (Dimitriadis et al. 2018) and that digital marketing analytics are inherently limited by the data they include (Kingsnorth 2019). Market research beyond what an organization's existing digital analytics tools can readily provide may be required, whether through traditional market research methods or a specially designed analytics project (Grigsby 2015; Van Bommel et al. 2014). Column three focuses attention on selecting specific and contextually appropriate digital marketing channels and analytics tools, a key task of digital marketing (Young 2014; Kingsnorth 2019). It must be informed by the segment and stage-specific value understanding developed in the prior column.

**Table 1:** Creating consumer value: using the consumer decision journey for strategic selection of digital marketing analytics tools

Stage	Tools to understand value	Tools to understand channel effectiveness	Tools to understand stage outcome
See	Use quantitative *and* qualitative research to understand the value for our segment	Use channel-specific metrics to measure success in communicating our value to our segment	Measure of awareness
Think			Measure of active evaluation
Do			Measure of purchase
Care			Measure of loyalty

The goal of this framework is to ground the digital marketer’s selection of channels and their corresponding digital marketing analytics in service to consumer’s needs, and in so doing, integrate these decisions with an organization's marketing strategy. The framework is intended to be used for analyzing the decision journey of *each* segment that an organization has targeted. It is *not* intended to prescribe specific digital marketing analytics techniques or tools, but rather to ensure the techniques and tools that are selected are strategically sound and not a quick path to digital marketing analytics myopia.

To better illustrate the value of the proposed framework we will apply it to the hypothetical example of an athletic apparel company with a target segment of basketball players. Digital marketers may find it helpful to translate the guidance of each row of the framework into a concise, template like statement:

- Our SEE stage goal is to increase our SHARE OF VOICE (Measure of Awareness) among BASKETBALL PLAYERS (Segment) by using INSTAGRAM INFLUENCERS (Channels) to position our brand as SOURCE OF CONFIDENCE ON AND OFF THE COURT (Value).
- Our THINK stage goal is to increase BRAND SEARCHES (Measure of Intent) among BASKETBALL PLAYERS (Segment) by using DISPLAY (Channels) to position our brand as HIGH QUALITY AND AFFORDABLE (Value).
- Our DO stage goal is to increase CART CONVERSION RATE (Measure of Purchase) among BASKETBALL PLAYERS (Segment) by using A CHATBOT (Channels) to OVERCOME DOUBTS ABOUT SIZES (Value).
- Our CARE stage goal is to increase REPEAT PURCHASE RATE (Measure of Loyalty) among BASKETBALL PLAYERS (Segment) by using BRAND APP (Channels) to position our brand as A VEHICLE FOR SELF-EXPRESSION (Value).

It is important to note that the value for each segment may slightly change from stage to stage. This is because consumers’ needs change slightly from stage to stage (Court et al. 2009). A consumer in the Think stage may need specific information, while a consumer in the Do stage may need specific reassurance. A consumer in the See stage may need to be introduced to the emotional benefits your brand provides, but a consumer in the Think stage may already be persuaded of these benefits and they are weighing your other product attributes against those of a competing brand that provides the same emotional benefits (Peter and Olson 2010). Digital marketers will not know exactly what a consumers’ stage needs are until they do the research to find out. Digital channel and analytics choices must always follow an understanding of a segment’s conception of value in each decision journey stage.

It is also important to note that although the hypothetical example only lists one channel choice for each stage, in practice there will be many channels employed. Each selected channel should have associated analytics, and none of those metrics alone will be able to determine if digital marketing efforts are successful or not. Limiting channel analytics until the meaning and effects of each are well understood would be wise. Evaluating channel analytics should be viewed as an equation:

$${\text{Value}}\;\left( {\mathbf{V}} \right) + {\text{Sum}}\;{\text{of}}\;{\text{Channel}}\;{\text{Effectiveness}}\;(\Sigma {\mathbf{ce}}) = {\text{Stage}}\;{\text{Outcome}}\;\left( {\mathbf{O}} \right).$$

If a digital marketer has put in the work to ensure a proper understanding of consumer value, then disappointing stage outcome results should be addressed by reconsidering and/or optimizing the digital marketing channels. However, if the digital marketer did not start by understanding value, value is where stage outcome problem resolutions must begin; to focus on channel effectiveness without first addressing value is to fall victim to digital marketing myopia.

Finally, just as this framework does not seek to prescribe specific digital analytics tools for understanding value or evaluating channel effectiveness, it is not prescribing specific methods for measuring stage outcomes. These should be selected based on an individual organization’s expert knowledge of their segments, industry, and overall marketing strategy.

## Limitations and future research

The strategic framework proposed in this paper is limited in several ways. First, it is merely conceptual and has not been tested by practitioners or researchers. It also assumes that an organization has already invested significant effort in developing a marketing strategy. It assumes that an organization has identified clear target segments, has tailored its offerings to the needs of those segments and has communicated this information across departmental silos. Perhaps most importantly, we have not attempted to address the appropriate timeframes and stage segmentation procedures for implementing the framework. How often should digital marketers go through the process recommended by the framework, and when they select that timeframe, is it necessary to measure stage outcomes in a way that isolates consumers according to their stage during the time considered? In order to bridge the gap between theory and practice, the testing and application of this construct are essential by future researchers to gain a greater understanding of the consumer decision journey in the world of digital marketing analytics.

## Conclusion

As marketing evolves toward an increasingly digital future, the field of marketing analytics will be challenged to make sense of more data from more diverse sources. However, technology and relevant marketing channels may change, the consumer decision journey is a strategic marketing framework that can guide digital marketing analytics practitioners and academics toward contributions that create real value for consumers and firms. Mapping the consumer decision journey helps marketers understand what consumers want and how to connect them to it. The process can illuminate consumer needs and expectations, informing content creation (Malthouse et al. 2016), UX design, and marketing channel choices. This framework provides strategic guidance to avoid marketing reactions that are misaligned with organizational and consumer expectations. As marketers gain deeper insight into these specifics of consumer decision journeys, they should beware of the temptation to unleash ever more analysis and KPIs; the proliferation of these is not a strategy, but a quick path to digital marketing analytics myopia. Instead, savvy marketers will leverage analytics at each stage of the consumer decision journey to meet consumers' needs and align digital and firm-level marketing strategy.
